# Multiple Targeting Approaches on Histamine H_3_ Receptor Antagonists

**DOI:** 10.3389/fnins.2016.00201

**Published:** 2016-05-30

**Authors:** Mohammad A. Khanfar, Anna Affini, Kiril Lutsenko, Katarina Nikolic, Stefania Butini, Holger Stark

**Affiliations:** ^1^Stark Lab, Institut fuer Pharmazeutische and Medizinische Chemie, Heinrich-Heine-Universitaet DuesseldorfDuesseldorf, Germany; ^2^Faculty of Pharmacy, The University of JordanAmman, Jordan; ^3^Department of Pharmaceutical Chemistry, Faculty of Pharmacy, University of BelgradeBelgrade, Serbia; ^4^Department of Biotechnology, Chemistry, and Pharmacy, European Research Centre for Drug Discovery and Development, University of SienaSiena, Italy

**Keywords:** multiple targeting, GPCR, enzymes, NO, histamine, transporter

## Abstract

With the very recent market approval of pitolisant (Wakix®), the interest in clinical applications of novel multifunctional histamine H_3_ receptor antagonists has clearly increased. Since histamine H_3_ receptor antagonists in clinical development have been tested for a variety of different indications, the combination of pharmacological properties in one molecule for improved pharmacological effects and reduced unwanted side-effects is rationally based on the increasing knowledge on the complex neurotransmitter regulations. The polypharmacological approaches on histamine H_3_ receptor antagonists on different G-protein coupled receptors, transporters, enzymes as well as on NO-signaling mechanism are described, supported with some lead structures.

## Introduction

The idea of synthesizing multiple targeting compounds arises from the fact that the paradigm “one drug—one target” or “single-target drug” is not sufficiently meeting the need for the treatment of a large number of complex diseases caused by multifunctional pathophysiological processes. Since central nervous system (CNS) disorders are characterized by diverse physiological dysfunctions and deregulations of a complex network of signaling pathways, optimal multipotent drugs should simultaneously and specifically modulate selected groups of biological targets. Polypharmacology is a new scientific area focused on discovery, development, and pharmacological study of Multiple Targeting Designed Ligands (MTDL) able to simultaneously modify the activities of several interacting pharmacological targets (Hopkins, 2008).

This emerging approach suggests that multifactorial CNS diseases such as depression (Millan, 2014), schizophrenia (Ye et al., 2014), Parkinson's disease (PD) and Alzheimer's disease (AD; Youdim and Buccafusco, 2005; Leon et al., 2013) can be treated with higher efficacy, lower toxicity, less drug-drug interactions, and also with unified pharmacokinetic profile if a single drug molecule is able to simultaneously interact with multiple targets (Anighoro et al., 2014; Huang et al., 2015).

**Figure 1:**
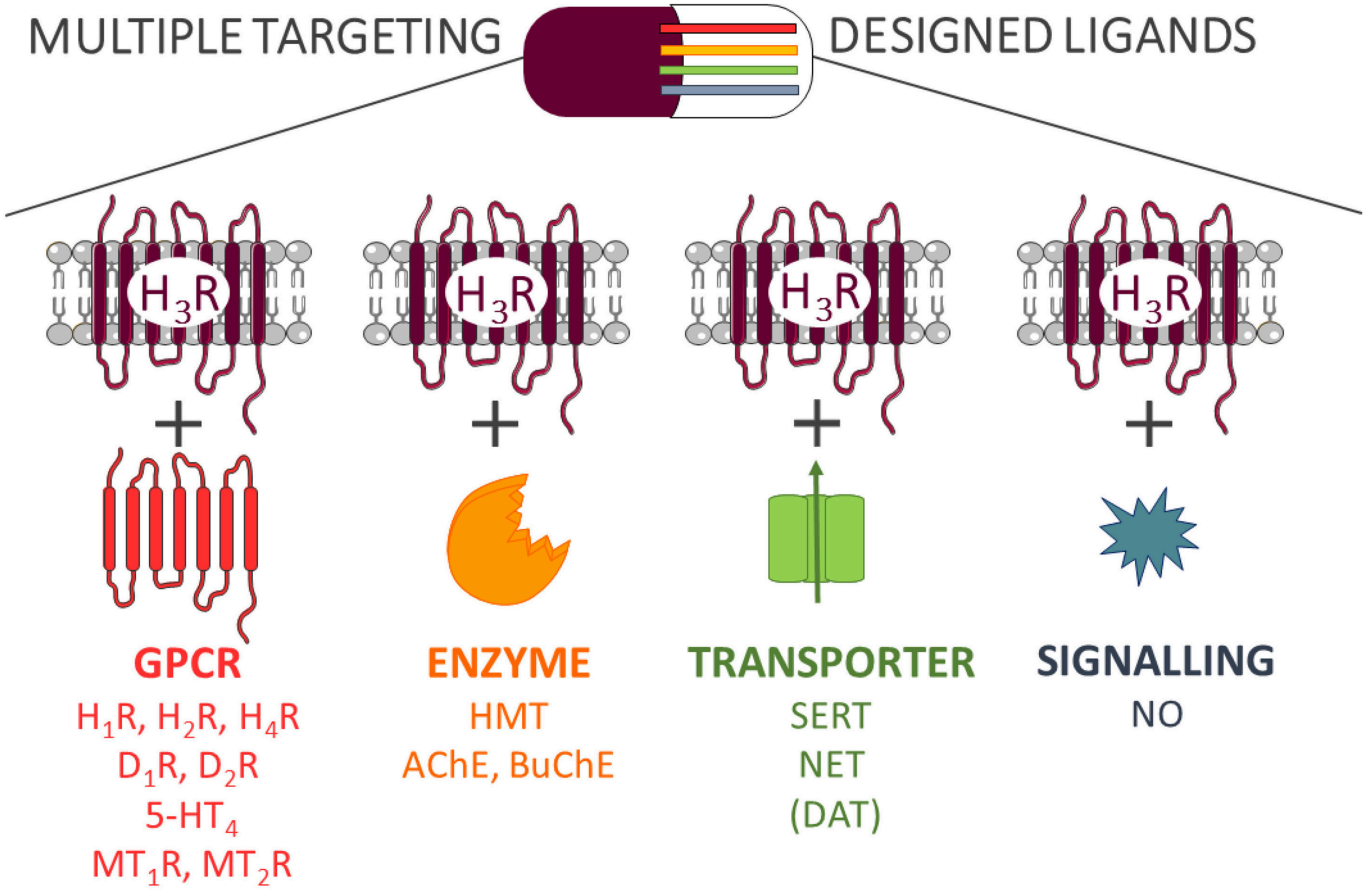
**Multi-targeting designed ligands with H_3_R**.

Despite the positive effects of MTDL, there are several potential disadvantages, which need to be taken into consideration. In order to identify multiple targeting hits, a more detailed and extensive pharmacological characterization of current drug-target interactions is needed (Peters, 2013). In most previous cases, the need for a polypharmacology to reach a therapeutic effect is discovered retrospectively. After finding a lead compound for a specific group of targets, the optimization of complex structure-activity relationships (SAR) profile is one of the first challenging tasks from a medicinal chemistry point of view. Most importantly, simultaneous targeting of several receptors may lead to a wider and sometimes unpredictable spectrum of biological activities such as side effects. Therefore, a balance between polypharmacological benefits and potential drawbacks brought by promiscuous scaffolds needs to be evaluated at least as carefully as with all other candidates, but based on a more complex behavior (Anighoro et al., 2014). Herein we describe the current implementation of target-oriented polypharmacological approaches with histamine H_3_ receptor (H_3_R) ligands based on research findings (Figure 1).

H_3_R is a member of transmembrane class A of G protein–coupled receptors (GPCR) family (Arrang et al., 1983; Schwartz et al., 1991). It influences several intracellular pathways through its coupling to Gα_i/o_ (Bongers et al., 2007). Analysis of H_3_R mRNA in rat (Héron et al., 2001) and human (Jin and Panula, 2005) brains showed that H_3_R is largely expressed on the histaminergic neurons of the CNS (located presynaptically and postsynaptically; Jadhav and Singh, 2013). As auto-receptor, H_3_R plays an important role in histamine biosynthesis and release and as hetero-receptor in the modulation of different neurotransmitters release (e.g., acetylcholine, noradrenaline, dopamine, GABA, glutamate, and serotonin; Schlicker et al., 1989, 1990). A lower level of H_3_R is distributed in the peripheral nervous system and is responsible for the regulation of sympathetic effector systems and pain sensation (Héron et al., 2001). Therefore, modulation of the H_3_R can potentially prevent the activation of the negative feedback mechanism leading to increased neurotransmitter release. Consequently, targeting of H_3_R with antagonist/inverse agonist may have therapeutic applications in CNS-related disorders, such as depression, schizophrenia, PD, and AD (Esbenshade et al., 2008; Gemkow et al., 2009; Chazot, 2010; Raddatz et al., 2010; Lin et al., 2011; Ghasemi and Tavakoli, 2012) as well as in inflammatory and gastrointestinal diseases (Vuyyuru et al., 1995; Ceras et al., 2012). Recently, several substances have entered late clinical phases for the treatment of several CNS disorders (Sander et al., 2008; Panula et al., 2015).

## H_3_R/H_1_R

The drug Betahistine (*N*-methyl-2-(2-pyridyl)ethanamine), indicated for the treatment of vestibular Morbus Menière, can be considered as the first MTDL in this category by working as an agonist at histamine H_1_ receptor (H_1_R) and antagonist at H_3_R (Lian et al., 2014, 2016; Møller et al., 2015). The H_3_R antagonism leads to inhibition of vestibular neurotransmission, central vasodilatation with potential antipsychotic effects, whereas the H_1_R agonism have an immune-regulatory effect (Dagli et al., 2008; Zhou et al., 2013).

Currently, the main focus on polypharmacological targeting of H_3_R/H_1_R is to develop dual agonist or dual antagonist ligands. Dual acting H_3_/H_1_ receptor (H_3_R/H_1_R) antagonists were synthesized for the treatment of allergic diseases. These diseases are associated with the degranulation of the mast cell and histamine release which can activate H_1_R and consequently stimulates phospholipase C that ultimately liberate inositol-1,4,5-trisphosphate and Ca^2+^; thereby improves mucus secretion and vasodilatation (McLeod et al., 1999; Bakker et al., 2002).

H_1_R antagonists play a key role in the treatment of allergic rhinitis; however, there are several limitations to their clinical use. The first generation of H_1_R antagonists (e.g., Diphenhydramine, Chlorpheniramine) show sedative effects whereas second generation H_1_R blockers (e.g., Loratadine, Mizolastine) have poor penetration to the CNS, thus generating non-sedating antihistaminic activity (Cowart et al., 2004; Stark et al., 2004). However, the second generation H_1_R blockers are often combined with α-adrenergic agonists to stimulate normal vascular tone and to reduce nasal congestion. Such combination is associated with serious cardiac side effects (QT time prolongation, ventricular arrhythmia).

These findings have encouraged several research groups to consider if other histamine receptor subtypes may contribute to the histamine-induced nasal congestion. Several studies confirmed that H_3_R may play an important role in histamine-induced nasal congestion because the vasodilatation is caused by activation of H_3_R in peripheral post-sympathetic ganglionic neurons (Hey et al., 1992). The activation of the H_3_R hetero-receptors located on neighboring noradrenergic neurons (Berlin et al., 2011) modulates the release of the neurotransmitter noradrenaline in the nasal blood vessel. Therefore, a compound that antagonizes H_1_R on one hand and inhibits H_3_R on the other hand may treat allergic diseases without having nasal congestion.

**Figure 2:**
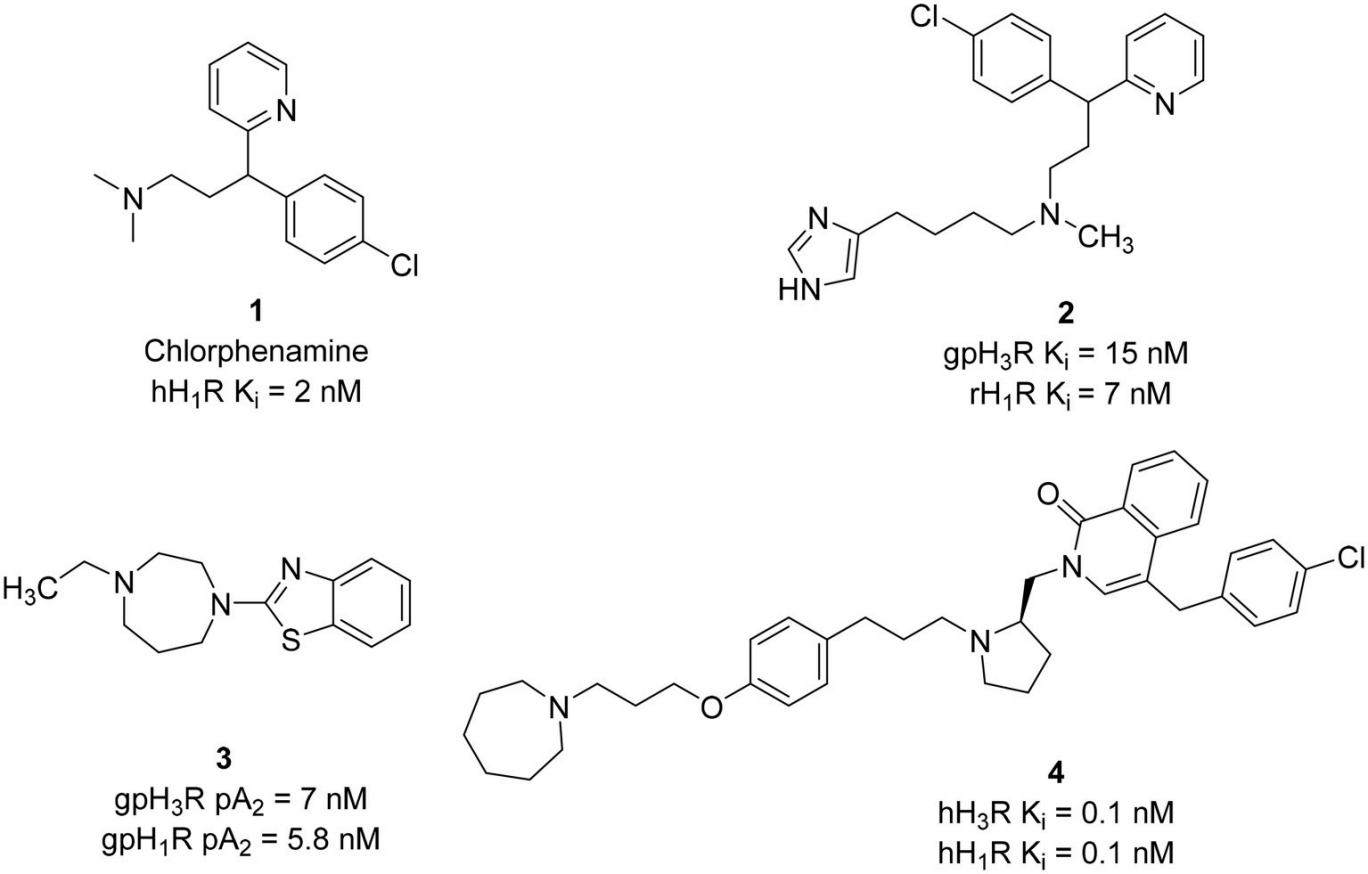
**Structures and biological activities of selected H_3_R/H_1_R ligands**.

Based on first and second generations of H_3_Rantagonists, imidazole and non-imidazole H_3_R/H_1_R ligands were designed. Several imidazole-derivatives taking Chlorphenamine **1** (hH_1_R K_i_ = 2 nM) as an additional pharmacophore for the introduction of H_1_R antagonist activity show dual H_3_R/H_1_R inhibitory affinity (Wieland et al., 1999). Limited variations of the linker in both sides of the aliphatic amino moiety provided compounds with good H_3_R binding affinity. Like all aminergic GPCR, H_1_R, and H_3_R contain an aspartate residue in the transmembrane domain III, that is involved in electrostatic interaction with protonated amino functionality (Wieland et al., 1999). Therefore, replacement of the basic amino linker by a neutral linker such as amide or urea, resulted in activity loss on the H_1_R. However, incorporating a tertiary amine led to the synthesis of the most potent dual inhibitor in that series (compound **2**, Figure 2) that displays affinities at low nanomolar concentration range for both H_1_R and H_3_R.

Further structural optimization was conducted by replacing the imidazole ring with different heterocycles in order to avoid potential interactions with CYP450 enzymes. In one of the trials, the non-imidazole heterocycles were combined with a benzothiazole structure (Walczyński et al., 1999). *In vitro* results of this series from guinea pig ileum system showed increasing H_3_R antagonist potency in the presence of an alkyl-substituted azepane (compound **3**, Figure 2). However, this compound showed weak H_1_R antagonist activity, with pA_2_ value of 5.77. A similar approach was applied in designing H_3_R/H_1_R dual inhibitors by combining nitrogen-containing heterocycles, with a benzylphthalazinone (GSK-1004723), compound **4** (Figure 2), or a quinoline structure (GSK-835726) (Slack et al., 2011; Daley-Yates et al., 2012), and WO-094643 (Norman, 2011). Compounds **4** and GSK-835726 were potent H_3_R/H_1_R antagonists *in vitro* and *in vivo* systems. Compound 3 has a major advantage associated with its long duration of action (t_1/2_ of 1.2–1.5 h, Table 1) which allows once a day intranasal dosing for the treatment of allergic rhinitis. GSK–1004723 completed phase II of clinical trials for the treatment of allergic rhinitis.

**Table 1:** **Selected pharmacokinetic data of preclinical candidates (Ly et al., 2008; Slack et al., 2011; Daley-Yates et al., 2012)**.

**Code**	**C_*max blood*_**	**C_*max brain*_**	***t*_0.5_**	***F***	***Cl***	**Vss**	**K_*off*_**	**K_*on*_**
**4**	–	–	1.2–1.5 h	–	–	–	0.007 ± 0.001 min^−1^	4.76 ±0.69 × 10^8^
**GSK-835726**	0.747 μM	–	15.5 h	–	–	–	0.802 ± 0.010 min^−1^	3.04 ±0.14 × 10^9^
**26**	1 μM	1 μM	16.1 ± 0.9 h	93%	10.2 ± 1.0 mL/min/k	13.0 ± 1.2 L/kg	–	–

## H_3_R/H_2_R

**Figure 3:**
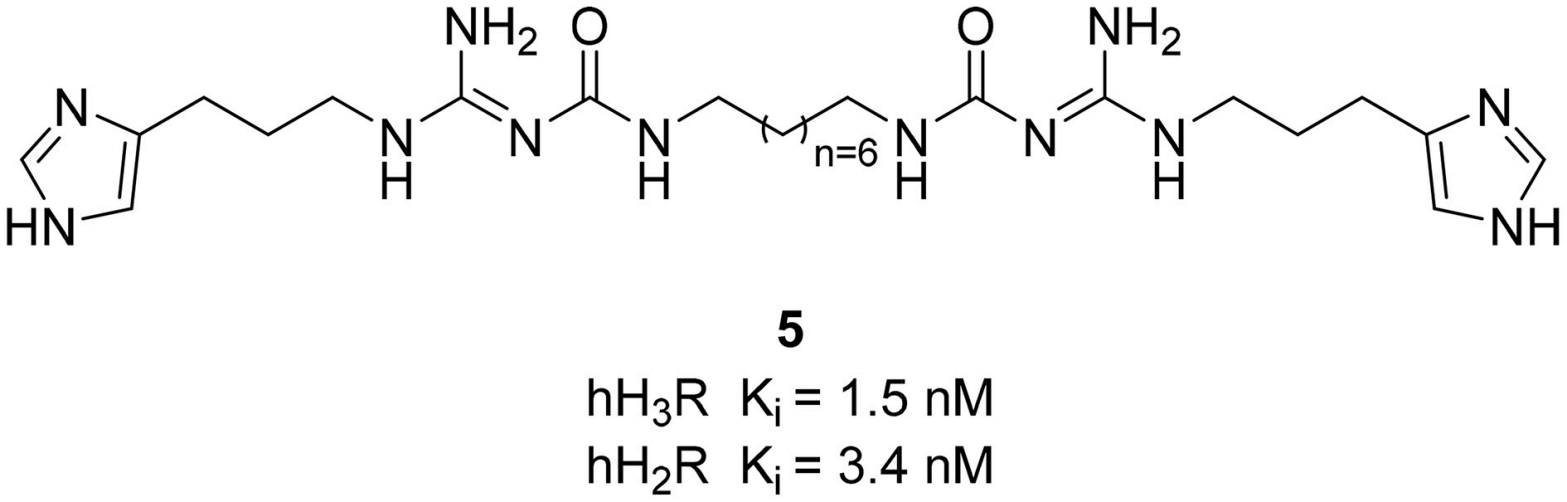
**Structures and biological activities of selected H_3_R/H_2_R ligands**.

Limited efforts have been conducted so far for the designing of dual H_3_R/H_2_R ligands. However, guanidine-based histamine H_2_R ligands demonstrate additional H_3_R antagonist potencies. Recently, Buschauer et al. investigated dimeric carbamoylguanidine derivatives for the synthesis of potent H_2_R agonists (Kagermeier et al., 2015). Compounds containing two imidazole moieties, display selectivity for H_3_R and H_2_R in radioligand competition binding studies, whereas compound **5** (Figure 3) shows high H_2_R affinity with simultaneous high H_3_R inhibitory affinity. Since the brain penetration of these compounds is quite low, they can mostly be used on cells and isolated tissues.

## H_3_R/H_4_R

**Figure 4:**
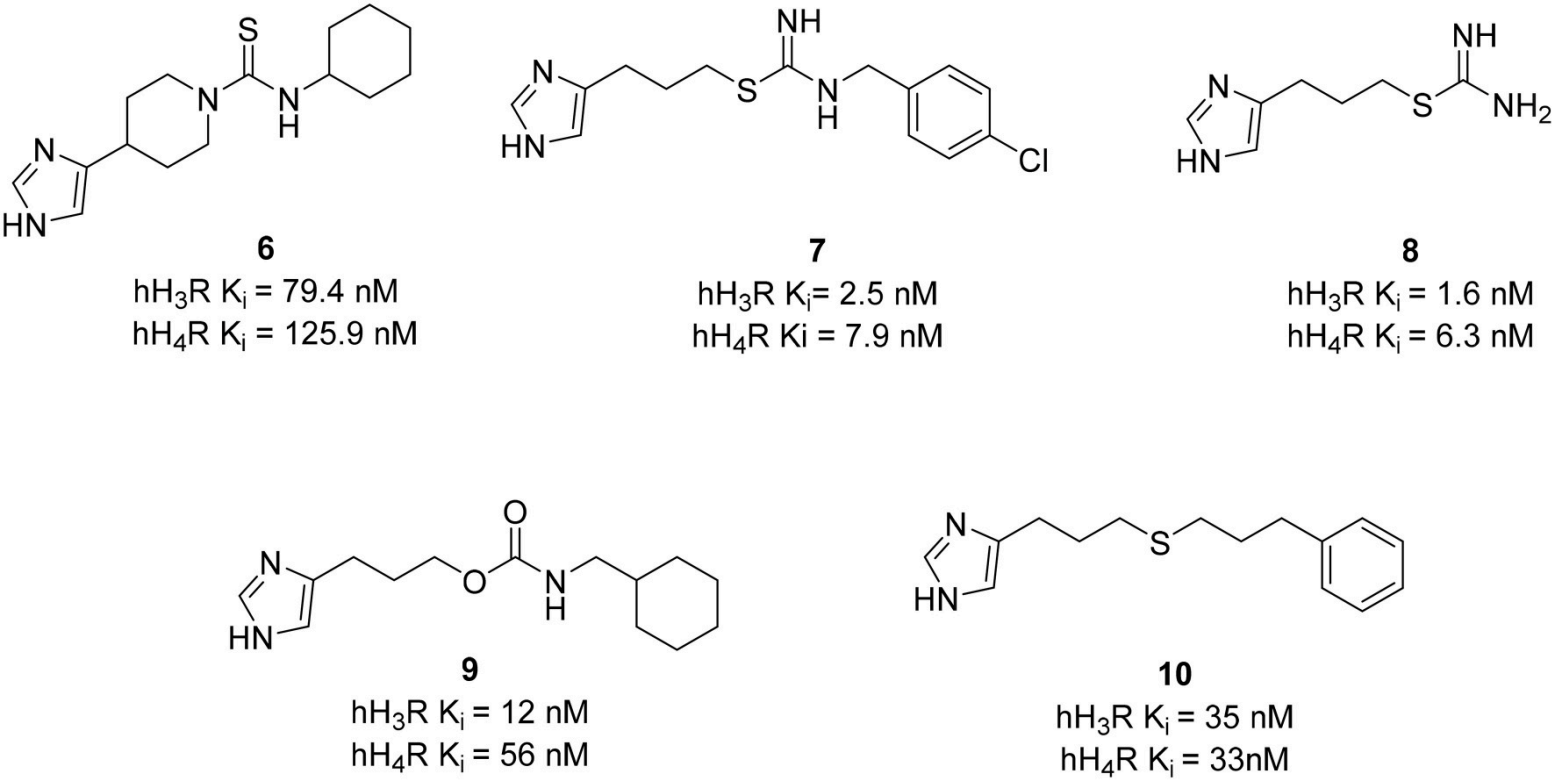
**Structures and biological activities of selected H_3_R/H_4_R ligands**.

Similarly, dual targeting is also often applied on histamine H_3_R and H_4_ receptors (H_4_R). Because of the relative high H_4_R homology with H_3_R (37% in entire sequence, 68% within transmembrane domains) many potent histamine H_3_R ligands containing imidazole moieties (**6–8**; Figure 4) show off-target affinity at H_4_R (Neumann et al., 2013). The human H_4_R is the last receptor subtype that has been identified in the histamine receptor family (Corrêa and Fernandes, 2015). The H_4_R is mainly located on cells of hematopoietic origin and, therefore, may be a promising target for the treatment of inflammatory diseases like allergic rhinitis, asthma, and pruritus (Thurmond et al., 2008). The expression of H_4_R in the CNS is a controversial topic because immunostaining methods are critically discussed and inconsistent mRNA screening results were obtained (Panula et al., 2015). Dual H_3_R/H_4_R ligands could be promising targets for pain and cancer since it is likely that these two targets contribute to the development of pain sensation and itching as well as cell-proliferation-associated effects (Medina and Rivera, 2010). However, further investigation is required to fully understand and evaluate their functions for therapeutic applications.

Clobenpropit (**7**), a potent reference H_3_R antagonist, was identified as a template for dual H_3_R/H_4_R ligands. Variations in substituents of the phenyl moiety as well as in the length of the alkyl chain between the central core isothiourea and the lipophilic aromatic residue were performed (Lim et al., 2009). Elongation of the spacer and introduction of bulky groups in the east part of these molecules such as diphenyl residue led to moderate affinity for both H_3_R and H_4_R. Nevertheless, most of these compounds showed moderate to high affinity at both H_3_R and H_4_R in a similar concentration range [human H_3_ receptor (hH_3_R) K_i_=2.5–79.4, hH_4_R K_i_= 1.6–158.4]. Compounds with a halogen substituent at the 4-position of the benzyl moiety showed the best binding affinities at both receptors. Further structural modifications were performed to expand the SAR on imidazole-containing histamine receptor ligands. Changing the polarity of the central core isothiourea by introducing different moieties such as amide, carbamate, urea, ester, ketone, and ethers was exploited (Kottke et al., 2011). Amide derivatives were unsuccessful because they had poor affinity at the hH_3_R. In contrast, all the other moieties bound to both receptors in a comparable concentration range, showing that these central cores of the alkyl imidazole can be used as a lead structure for dual acting H_3_R/H_4_R ligands. Among the carbamate series, the presence of a cycloalkyl moiety in the east part is important to have K_i_ values for both receptors below 200 nM. Cyclohexylmethyl derivative **9** (Figure 4) is the most potent H_3_R/H_4_R antagonist in that series (Wicek et al., 2011). It must be stressed that the affinity is not the only criteria for the MTDL selection. Some compounds may have similar affinities, but different efficacies. In this respect, replacing the carbamate function with a thioether group led to the synthesis of a potent dual H_3_R antagonist and H_4_R partial agonist **10** (Figure 4). These compounds are potent dual H_3_R/H_4_R ligands that can be optimized for further pre-clinical trials; however, no further work has been reported. Therefore, efficacy and not only affinity data has to be considered for the pharmacological profile evaluation of new drugs.

## H_3_R and non-histaminergic GPCRs

**Figure 5:**
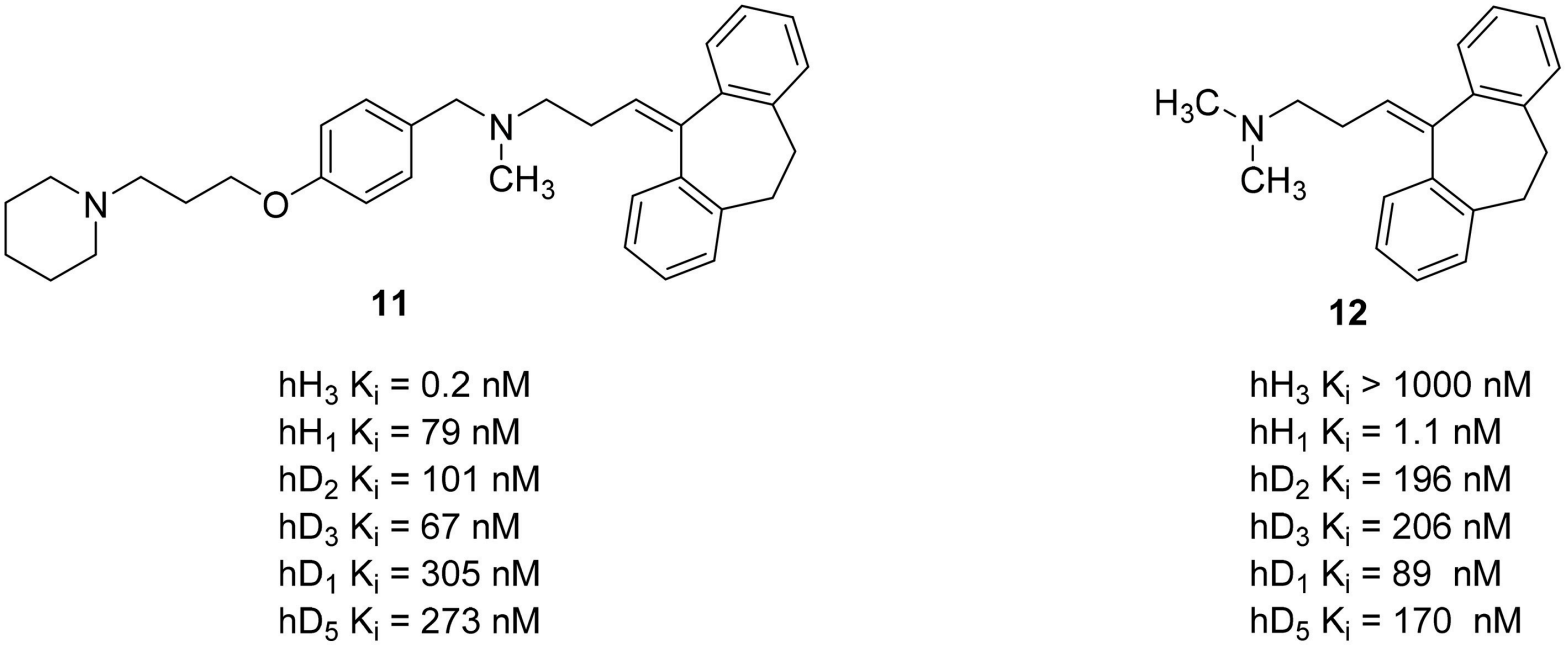
**Structures and biological activities of selected H_3_R/D_2_R ligands**.

In addition to combined properties with other histamine receptor subtypes, other aminergic GPCRs have also been addressed with polypharmacological targeting of H_3_R. Dopamine is an important neurotransmitter in the human brain. It affects almost all mental functions, such as movement control, motivation, emotion, learning, and memory. Dysregulations of dopamine neurotransmitter system of the CNS may cause schizophrenia and related mood disorders (Schlicker et al., 1993; Witkin and Nelson, 2004). Neuroleptics used for the treatment of schizophrenia usually inhibit dopamine D_2_-like receptors and other aminergic receptors, such as serotonin 5-HT_2_A receptor, dopamine D_1_ receptor (D_1_R) receptors, and other serotonin receptor subtypes (Remington, 2003). The most important side effects of these neuroleptics are extrapyramidal side effects and weight gain problems (Vuyyuru et al., 1995; Deng et al., 2010; Lian et al., 2016). These side effects are related to their antagonistic properties at the dopamine D_2_-like and H_1_R, respectively (Kroeze et al., 2003; Von Coburg et al., 2009). Additionally, schizophrenic patients usually showed a significantly high level of *N*-methylhistamine in cerebral cerebrospinal fluid (Ligneau et al., 2007). There are several studies showing an interaction between histamine H_3_R and dopamine D_2_ receptors (D_2_R) as well as H_3_R and D_1_R as oligomeric hetero-receptors (Humbert-Claude et al., 2007; Ferrada et al., 2008). Furthermore, H_3_R inverse agonists/antagonists showed a reduction of undesirable side effects like weight gain, somnolence, and cognitive impairment in several rodent models of schizophrenia while displaying a significant inhibitory activity (Ligneau et al., 2007). Combining the known H_3_R antagonists pharmacophore 4-(3-piperidinopropoxy)phenyl with known neuroleptics may provide novel multi-acting antipsychotic drugs with an improved pharmacological profile and reduced side effects by decreasing H_1_R affinity and introducing H_3_R activity while maintaining D_2_R/D_3_R affinity (Humbert-Claude et al., 2007; Von Coburg et al., 2009). For this approach 4-(3-piperidinopropoxy)phenyl was linked to several known neuroleptics. Resulting compounds showed high H_3_R affinity with K_i_ values between 4.90 nM and 42 pM while simultaneously reduced the H_1_R affinity by a factor of 10–600 as off-target and maintained the D_2_-like receptor subtypes affinity (Figure 5; Deng et al., 2010). Compound **11** (Figure 5) with a good overall profile and high H_3_R affinity was synthesized by merging 4-(3-piperidinopropoxy)phenyl fragment with amitriptyline **12** (Figure 5). This compound was selected for an early *in vivo* screening for central H_3_R antagonist potency on male Swiss mice. To determine the *in vivo* potency, an increase in *N*-methylhistamine level in the brain 90 min after the oral application of the compound was measured (Von Coburg et al., 2009). Unfortunately, this compound seems to be inactive (ED_50_ > 10 mg/kg p.o.) with unclear reasons mostly for absorption, distribution, or metabolism.

**Figure 6:**
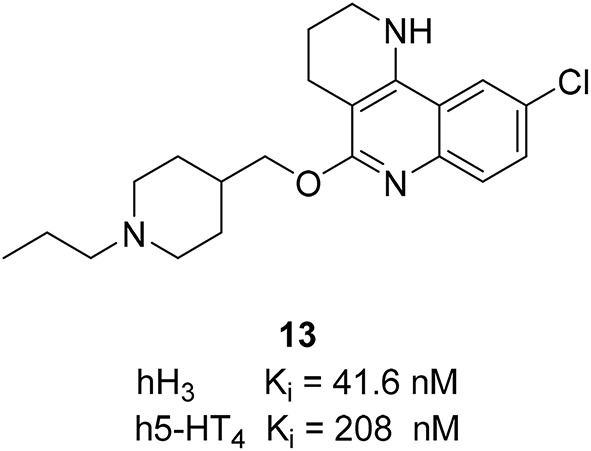
**Structure and biological activity of the selected H_3_R/5-HT_4_ ligand**.

Using pharmacophore-based virtual screening, Lepailleur et al. identified an interesting additional target activity while analyzing the screening hits (Lepailleur et al., 2014). A series of tricyclic derivatives have high serotonin 5-HT_4_ receptor (5-HT_4_R) affinity. There is a connection between different serotonin receptor subtypes, especially on 5-HT_1A_R, 5-HT_4_R, and 5-HT_6_R and emerging AD therapies (Sabbagh, 2009; Mangialasche et al., 2010; Herrmann et al., 2011) and other degenerative disorders connected to an impaired cholinergic function (Esbenshade et al., 2008; Sander et al., 2008; Gemkow et al., 2009). 5-HT_4_R provide symptomatic alleviation of cognitive impairments and neuroprotection by reducing amyloid-β (βA) generation and toxicity (Lezoualc'h, 2007). 5-HT_4_R activation improves cognitive processes such as learning and memory (Lelong et al., 2001, 2003; Levallet et al., 2009; Hotte et al., 2012). Combined with the beneficial effects of H_3_R on neurodegenerative diseases, dual targeting of H_3_R and 5-HT_4_R would therapeutically be useful. One of the identified hits, compound **13** (Figure 6) showed high affinities with K_i_ values of 41.6 nM at H_3_R and 208 nM at 5-HT_4_R and significant selectivity over 5-HT_1A_R and 5-HT_6_R. Compound **13** was able to reverse the scopolamine-induced cognitive impairment partially at 1 mg/kg and completely at 3 mg/kg in a spatial working memory experiment (Klinkenberg and Blokland, 2011). Scopolamine is a nonselective muscarinic antagonist, which partially blocks the cholinergic neurotransmission and is used to examine the cognitive enhancing effects of potential compounds (Snyder et al., 2005; Fredrickson et al., 2008). These results reveal the potential of combined H_3_R antagonist/5-HT_4_R agonist profiles in one multi-targeting compound to modify symptomatic effects in Alzheimer's disease.

**Figure 7:**
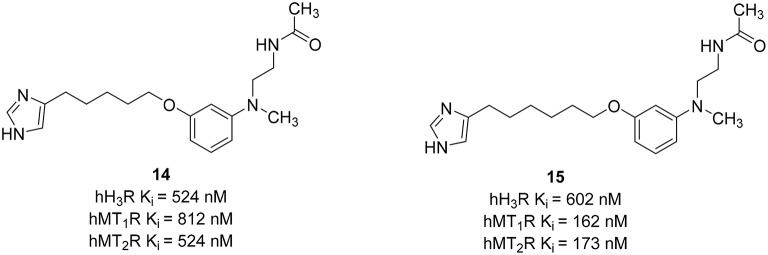
**Structures and biological activities of selected H_3_R/melatonin receptor ligands**.

Recently, different combinations between melatonin and another neuroprotection agent, e.g., curcurmin derivatives, have shown that melatonin may have a therapeutic potential in the treatment of cognitive disorders and neurodegenerative pathologies like AD (Chojnacki et al., 2014). Different H_3_R antagonists also showed neuroprotective actions (Brioni et al., 2011). Therefore, the synthesis of ligands able to bind at both H_3_R and melatonin receptors could be useful for the treatment of the diseases mentioned above. Pala et al. have synthesized compounds that can interact simultaneously with the H_3_R and melatonin T_1_ receptor (MT_1_R) and melatonin T_2_ receptor (MT_2_R; Pala et al., 2014). Melatonin is a methoxyindole-derived hormone secreted mainly by the pineal gland. The activation of MT_1_R and MT_2_R is not only important for the regulation of cardiac rhythms, but also for having antioxidant and neuroprotective effects (Srinivasan et al., 2006). For the synthesis of this melatonergic/histaminergic ligands the classical pharmacophore showed for potent H_3_R antagonists such as Ciproxifan and its analogs, was combined with an anilinoethylamide to have comparable binding affinity with the indol-3-ylrthylamide moiety of the melatonin (Figure 7). The length of the alkyl chain influences more the binding affinities at hMT_1_R and hMT_2_R than that at hH_3_R. Compounds with a short spacer such as a propyl or ethyl chain did not show affinity toward both MT_1_R and MT_2_R. One good dual acting ligand was obtained by elongating the alkyl chain between the imidazole ring and the melatonin moiety with a pentyl linker. The introduction of a six methylene unit improved the K_i_ values for both hMT_1_R and hMT_2_R. The elongation of the spacer can store the imidazole in a more peripheral region of the melatonin receptors. In that region, negative interactions with positively charged amino groups are weakened. Therefore, compounds (**14**, **15**; Figure 7) able to bind to both melatonin and histamine H_3_R with affinity in the micromolar concentration range were designed. The optimization of these ligands can be the next step for discovering new multiple targeting compounds that belong to the new melatonin-histamine combination.

## H_3_R and transporters

Selective serotonin reuptake inhibitors (SSRI) have been the drugs of choice to treat depression. However, the efficacy of these drugs is noticeable only after weeks of treatment and do not improve cognitive functions of depressive patients, which prompt many physicians to co-prescribe stimulants with SSRI to provide subjective relief. H_3_R antagonists produce wakefulness in animals without releasing dopamine or producing behavioral activation. Such activation has been avoided due to the risk of allowing patients to act on their suicidal ideation (Menza et al., 2000; Stahl, 2001). Combined H_3_R/SERT inhibition would provide symptomatic relief for the fatigue during the first weeks of treatment and afford immediate relief from some of the symptoms of depression with possible concurrent cognitive enhancement (Schlicker et al., 1998; Barbier et al., 2007; Nikolic et al., 2014).

**Figure 8:**
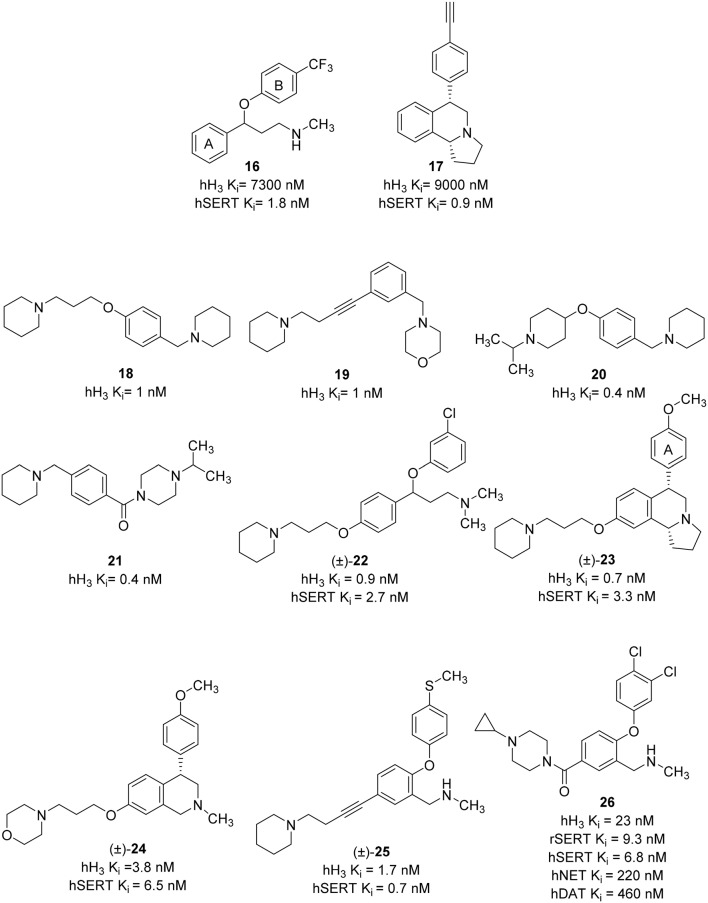
**Structures and biological activities of selected H_3_R/SERT ligands**.

Until now, most of the medicinal chemistry effort to develop new dual H_3_R/SERT inhibitors was conducted by Johnson & Johnson Pharmaceutical Research and Development group. Their effort was started with the identification of lead compounds with desirable SERT affinity, which could then be used as a template to introduce H_3_R antagonist activity. Two SSRI templates were designated, the first based on fluoxetine, which is the third most prescribed antidepressant drug (**16**, Figure 8; Wong et al., 1995), and the second based on the hexahydropyrroloisoquinoline scaffold represented by JNJ-7925476 (**17**, Figure 8), identified by high-throughput screening (Aluisio et al., 2008). Four templates of potent and selective H_3_R antagonists were considered to develop dual H_3_R/SERT inhibitors evaluated pre-clinically (**18**–**21**, Figure 8; Letavic et al., 2006). Starting from fluoxetine template, the tertiary benzyl amines of **18–21** were replaced with the fluoxetine template, so that the known SSRI would serve as both, the lipophilic core and one of the basic amines. Several H_3_ amine side moieties were initially 3- or 4-substituted on both phenylene rings of fluoxetine (rings A and B). All the regioisomers had high affinity for the hH_3_R, but the 3-piperidinyl-propyloxy derivative provided the highest affinity for both the rat serotonin transporter (rSERT) and human serotonin transporter (hSERT) (e.g., compound **22**, Figure 8; Stocking et al., 2007). The 4-(trifluoromethyl) substituted phenoxy (B) ring derivatives have no discrepancy between rSERT and hSERT, however, a decrease in affinity for hSERT over rSERT was observed for the unsubstituted derivatives. Electron donating substituents on B ring is associated with 5 to 30-fold decrease in hSERT affinity, however, electron withdrawing substituents displayed a good correlation between rSERT and hSERT (Stocking et al., 2007).

The same approach was applied for designing of hexahydropyrroloisoquinolines-derived dual H_3_R antagonists and SERT inhibitors. The overlap of the H_3_R antagonist **17** and SERT inhibitor **16** was pictured as exemplified in compound **23** (Figure 8). This approach generated a series of high affinity H_3_R antagonists with the SERT affinity dependent on aryl ring (A) substitution. Nevertheless, unlike the fluoxetine scaffold, most simple substitutions on the aryl ring (A) of the hexahydropyrroloisoquinoline scaffold provided similar rSERT and hSERT affinity (Keith et al., 2007c). On the other hand, the hydroxyl and the heterocyclic derivatives displayed a slightly higher affinity for rSERT than hSERT. Two high affinity compounds, the 4-methoxy derivative and the 3-pyridyl derivatives demonstrated good *in vivo* activities in serotonin potentiated head twitch model for SERT inhibition and blockade of imetit-induced drinking model for the H_3_R inhibition. However, this series showed unsatisfactory pharmacokinetics with low oral bioavailability, long t_1/2_ and a slow onset of action. In addition, these structures still retained affinity for the dopamine transporter (DAT; Keith et al., 2007c). Consequently, simpler templates from hexahydropyrroloisoquinoline were attempted, initially, by removal of the fused pyrrolidine ring and one chiral center to obtain the tetrahydroisoquinolines (Letavic et al., 2007a). Structural optimization of tetrahydroisoquinolines derivatives was conducted using a large number of amines in order to improve the binding affinity at H_3_R, varying the physical properties of the resulting compounds and maintaining SERT affinity (Keith et al., 2007b). Several modifications were attempted on the pendant piperidine ring; morpholine and substituted piperidines usually resulted in high affinity compounds. Replacing the piperidine with piperazine afforded compounds that have variable affinity for the hH_3_R, depending greatly on the basicity of the terminal nitrogen. For example, small alkyl substituents on the piperazine provided compounds with high affinity for the H_3_R, but decreasing the basicity of the terminal nitrogen by addition of bulky groups lowered the affinity for the H_3_R. Among the large number of derivatives that were synthesized, compound **24** (Figure 8), which was afforded by removal of the pyrrolidine ring of **23** together with the replacement of the piperidine ring with a morpholine, has improved rat pharmacokinetics and improved pharmacodynamics with a head twitch response (Keith et al., 2007a).

Further simplification was conducted by removing one carbon on the tetrahydroisoquinoline, which deleted the last remaining stereocenter to provide the benzyl amine derivatives (e.g., **25**, Figure 8). The benzylic carbon of tetrahydroisoquinolines was replaced with an oxygen in order to improve overall physical properties (Letavic et al., 2007b). The 3-piperidinyl-propyloxy derivatives were not used in this series; instead, they used the alkyne and amide side chains corresponding to the known H_3_R antagonists **19** and **21**. The later modification was important to avoid any potential metabolic problems associated with 1,4-hydroxyquinone. The SAR of alkynes was generally similar to that of the tetrahydroisoqinolines and most of the compounds have high affinity toward H_3_R and SERT. Selected compounds had good brain penetration in rat with brain levels of above 1 μM when dosed at 10 mg/kg p.o. (Letavic et al., 2007b). The benzamides benzyl amine derivatives were very potent with good selectivity over the norepinephrine transporter (NET) and DAT. One of the compounds, **26** (Figure 8), was extensively profiled *in vivo* and was found to have good rat pharmacokinetic and pharmacodynamics properties (Table 1; Ly et al., 2008). Although not yet tested on humans, inhibition of the H_3_R makes it an attractive combination with SERT blockade in order to create a novel antidepressant treatment.

**Figure 9:**
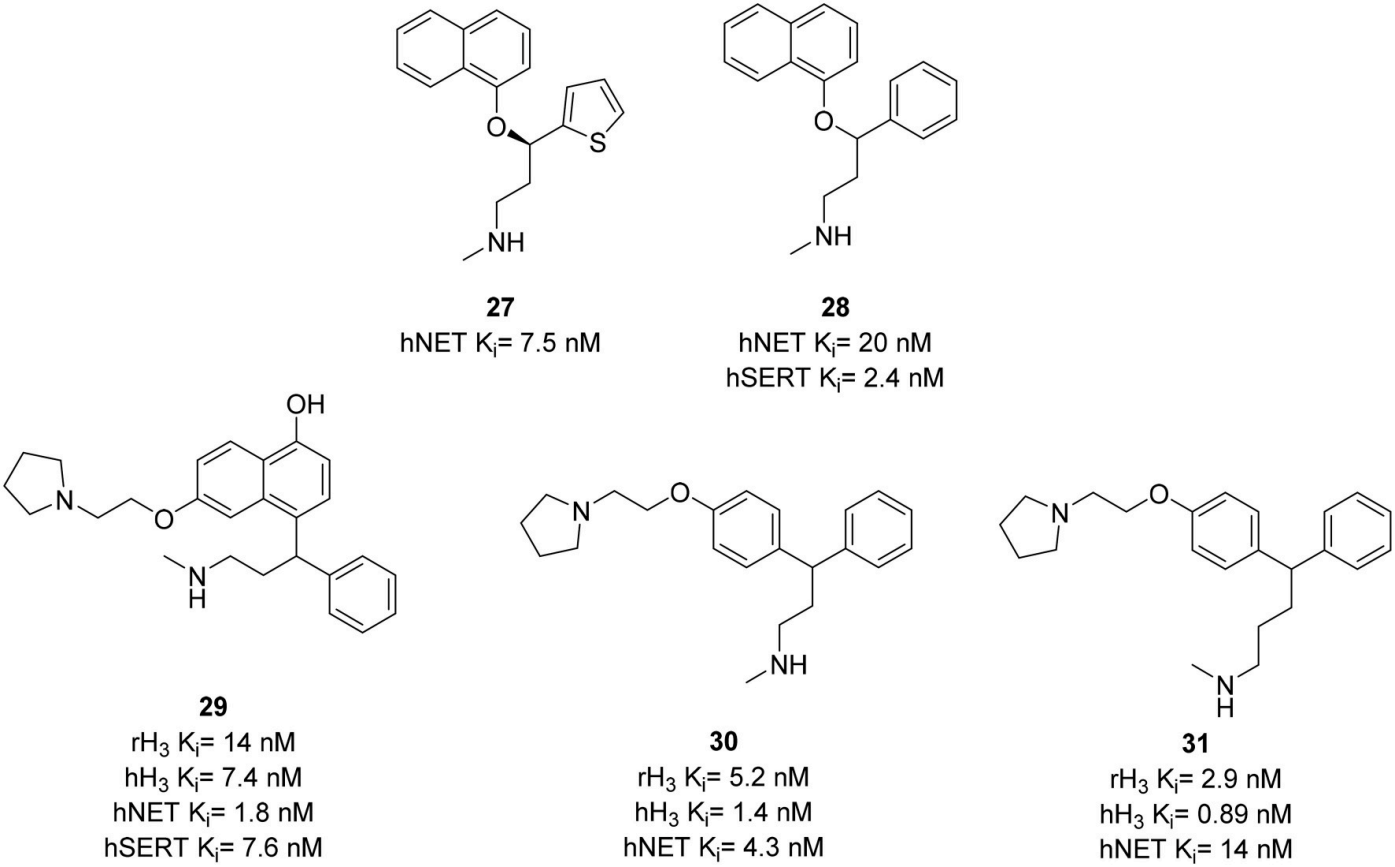
**Structures and biological activities of selected H_3_R/NET ligands**.

The serotonin/norepinephrine reuptake inhibitor (SNRI) duloxetine **27** (Figure 9) is used in therapeutic off-label treatment of neuropathic pain (Fishbain et al., 2006). The inhibition of NE uptake is essential for the pain efficacy (Leventhal et al., 2007). H_3_R antagonists Thioperamide **6** and GSK-189254 **28** (Figure 9) have been reported to be active in models of pain (Farzin et al., 1994; Medhurst et al., 2008). Using these results Altenbach et al. designed a series of molecules combining pharmacophores of H_3_R antagonism and NET inhibition in one molecule. An H_3_R pharmacophore was linked to duloxetine analogs, cf. **28** (Figure 9). Resulting compounds **29–31** (Figure 9) showed low nanomolar affinity at H_3_R and NET, where **29** additionally had SERT affinity (K_i_ = 7.6 nM) comparable to that of **28** (K_i_ = 2.4 nM; Bymaster et al., 2003). This affinity was reduced to K_i_ > 70 nM in compounds **30**, and **31** providing a better selectivity. Compound **29** was also found to be potent in osteoarthritis pain model in rats with efficacies of 70 and 93% at doses of 3 and 10 mg/kg, respectively (Anighoro et al., 2014).

## H_3_R and enzymes

Histamine level in the CNS is controlled not only by the receptors but also by the inactivating enzyme histamine *N*-methyltransferase (HMT; Parsons and Ganellin, 2006). Ligands with dual inhibitory activities on both H_3_R and HMT could increase intersynaptic histamine levels in the CNS and may lead to beneficial procognitive effects in psychiatric and neurodegenerative diseases (Apelt et al., 2002; Sander et al., 2008). Even if they have low or missing *in vivo* activity, such ligands could greatly enhance histaminergic neurotransmission *via* inhibition of histamine H_3_ auto-receptors and reduce the catabolic rate for histamine degradation *via* HMT inhibition (Grassmann et al., 2003).

**Figure 10:**
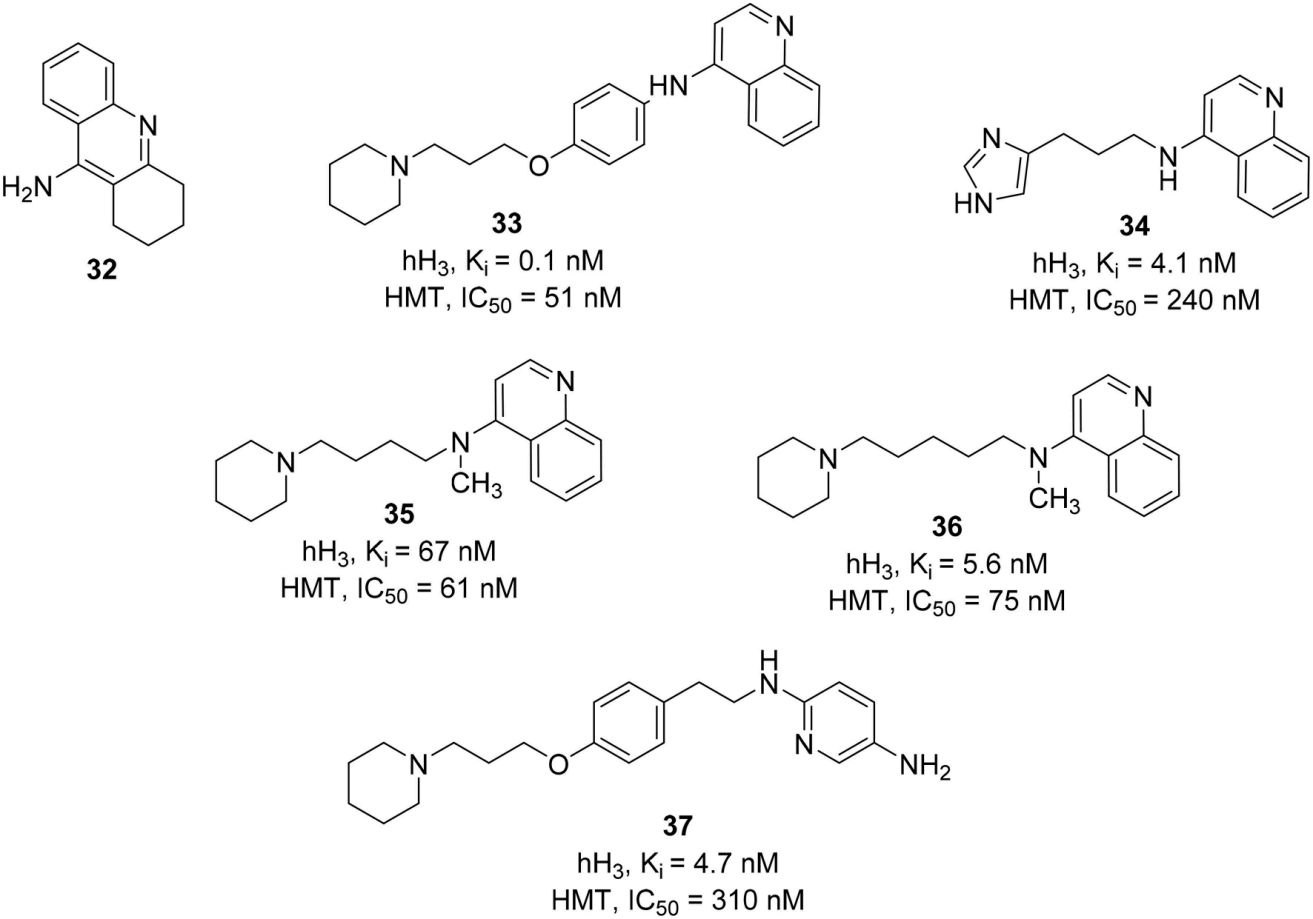
**Structures and biological activities of selected H_3_R/HMT ligands**.

Most of the HMT inhibitors have a 4-aminoquinoline moiety in common (e.g., tacrine, **32**, Figure 10). Therefore, the synthetic effort to develop novel and dual H_3_R\HMT inhibitors started from coupling of different 4-aminoquinolines with different spacers to the piperidine, the basic component that is essential for binding at the H_3_R. Variation of the spacer structure provides two different series of compounds. The first series have an alkylene spacer separating the basic center from the 4-aminoquinoline. These compounds showed potent HMT inhibitory activities with moderate to high H_3_R affinity. The second series, which possessed a *p*-phenoxypropyl spacer, showed a strong inhibitory activity on HMT and the H_3_R affinity, exceeding that of the first series. One of the compounds, FUB 836 (**33**, Figure 10), combines a high H_3_R affinity with a high HMT inhibitory activity and exhibited high H_3_R selectivity when compared to H_1_R and H_2_R (Apelt et al., 2002). Similar approach was applied in designing H_3_R/HMT dual inhibitors by combining imidazole heterocycle, which is an integral part of potent H_3_R antagonists, with several aromatic carbo- or heterocyclic structures (e.g., aminoquinoline or tetrahydroacridine moieties) of standard HMT inhibitors by different alkyl and alkenyl spacers. One interesting compound, **34** (Figure 10), showed a high H_3_R affinity with a high HMT inhibitory activity (Grassmann et al., 2003). Replacing imidazole head with a piperidine ring accompanied by a methylation of the amino functionality improved the inhibitory activity against HMT (e.g., compounds **35** and **36**; Figure 10; Grassmann et al., 2004).

Another approach was attempted on FUB 836 (**33**) by replacing the aminoquinoline with different heterocycles (e.g., nitro- or amino-substituted pyridines, quinolines, benzothiazole, or pyrroline) in order to improve its dual H_3_R/HMT affinities. In contrast to the aminoquinoline, the reported compounds showed moderate to good dual affinities. Whereas, some compounds showed potent HMT inhibitors, they only showed a moderate H_3_R affinity and *vice versa* (Apelt et al., 2005). The most potent compound in this series was 4-(3-piperidinopropyl)phenylether with substituted alkylaminopyridine (**37**, Figure 10).

Tacrine (**32**) mentioned above is an acetylcholinesterase (AChE) inhibitor. Together with the symptomatically acting *N*-methyl-D-aspartate (NMDA) blocker memantine, tacrine represents the only therapeutic treatment of AD currently available. AD is a complex neurodegenerative disorder and the most common form of dementia. Patients show a degeneration of cholinergic neutrons in the basal forebrain according to cholinergic hypothesis and aggregation of βA through an interaction with the peripheral anionic site (PAS) of the AChE (Davies and Maloney, 1976; Giacobini, 2000). H_3_R antagonists showed an ability to increase acetylcholine (ACh) but unlike the AChE, H_3_R antagonist will raise acetylcholine levels mostly in the brain, since H_3_R is mainly located in the CNS (Clapham and Kilpatrick, 1992; Darras et al., 2014). Therefore, the combination of both activities in a single molecule may offer the desired therapeutic effect with fewer unpleasant side effects considering acetylcholine release in the periphery (Fang et al., 2015; Guzior et al., 2015).

**Figure 11:**
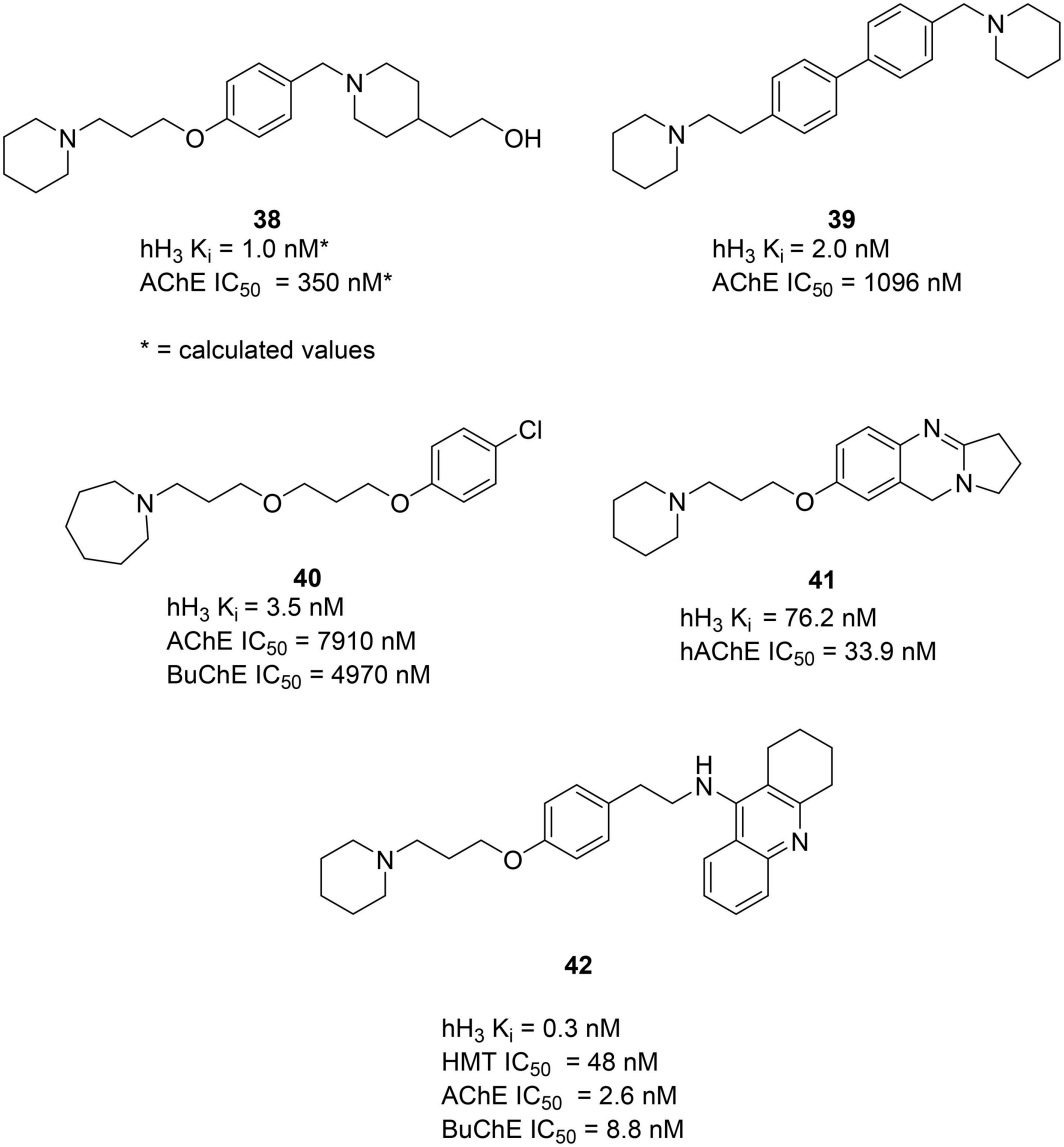
**Structures and biological activities of selected H_3_R/AChE ligands**.

Using available crystal structure information and applying pharmacophore modeling and docking simulations Bembenek et al. proposed compound **38** (Figure 11) and similar structures to have activity on both AChE and H_3_R. Moreover, the used models suggest a possible interaction for this series of compounds with the PAS of the AChE (Bembenek et al., 2008). Some additional *in vitro* an *in vivo* studies with these compounds could be of interest to verify the calculated results. In 2008 Morini et al. introduced a class of symmetric and asymmetric 4,4′−biphenyl H_3_R antagonists with a moderate ability to inhibit rat brain cholinesterase (Morini et al., 2008). This class is characterized by a rigid biphenyl scaffold and displays nanomolar binding affinities at human and rodent H_3_R. The compound **39** (Figure 11) showed low nanomolar affinity to the H_3_R and low micromolar activity to inhibit AChE. Docking the compound **39** into the catalytic cavity of mouse AChE showed similarity to the binding mode, earlier reported for **38**, confirming that more rigid and bulky biphenyl scaffolds are tolerated by the AChE active site. Interaction with PAS of the AChE is suggested for **39** as well as for **38**. In 2012 Bajda et al. presented a new class of diether derivatives of homo substituted piperidine with **40** (Figure 11) being the most active compound, showing low nanomolar affinity for the hH_3_R and micromolar inhibitory potency toward both cholinergic receptors (Bajda et al., 2012). In 2014 Darras et al. presented new tetracyclic nitrogen-bridge headed compounds showing balanced affinities as hAChE inhibitor and hH_3_R antagonist with UW-MD-71 (**41**, Figure 11). It showed the best activity in two digit nanomolar area for both targets and greater than 200-fold selectivity over the other histamine receptor subtypes. This compound was tested on acquisition, consolidation and retrieval in a model of dizocilpine-induced amnesia. Test results indicated that using multiple targeting ligands lead to pharmacological and behavioral profiles different from interaction with the respective single target ligands. Furthermore, a potential applicability in the modulation of the memory impairment could be shown (Khan et al., 2016).

In 2006, Petroianu et al. tested several compounds, containing structural features of tacrine (**32**) for their inhibitory activities on AChE and Butyrylcholinesterase (BuChE; Petroianu et al., 2006). These compounds have previously shown combined H_3_R antagonist and HMT inhibitory potencies (Apelt et al., 2002; Grassmann et al., 2003). From this series of compounds FUB833 (**42**, Figure 11) was the most promising four-target compound, showing subnanomolar affinity for hH_3_R, low nanomolar IC_50_ values for both cholinesterases and good affinity for HMT. These compounds have shown only moderate effects under *in vivo* conditions (Apelt et al., 2002). Furthermore, these new compounds might serve as novel important tools for further pharmacological investigations on histaminergic neurotransmission and its regulatory processes.

## H_3_R and no-releasing molecules

**Figure 12:**
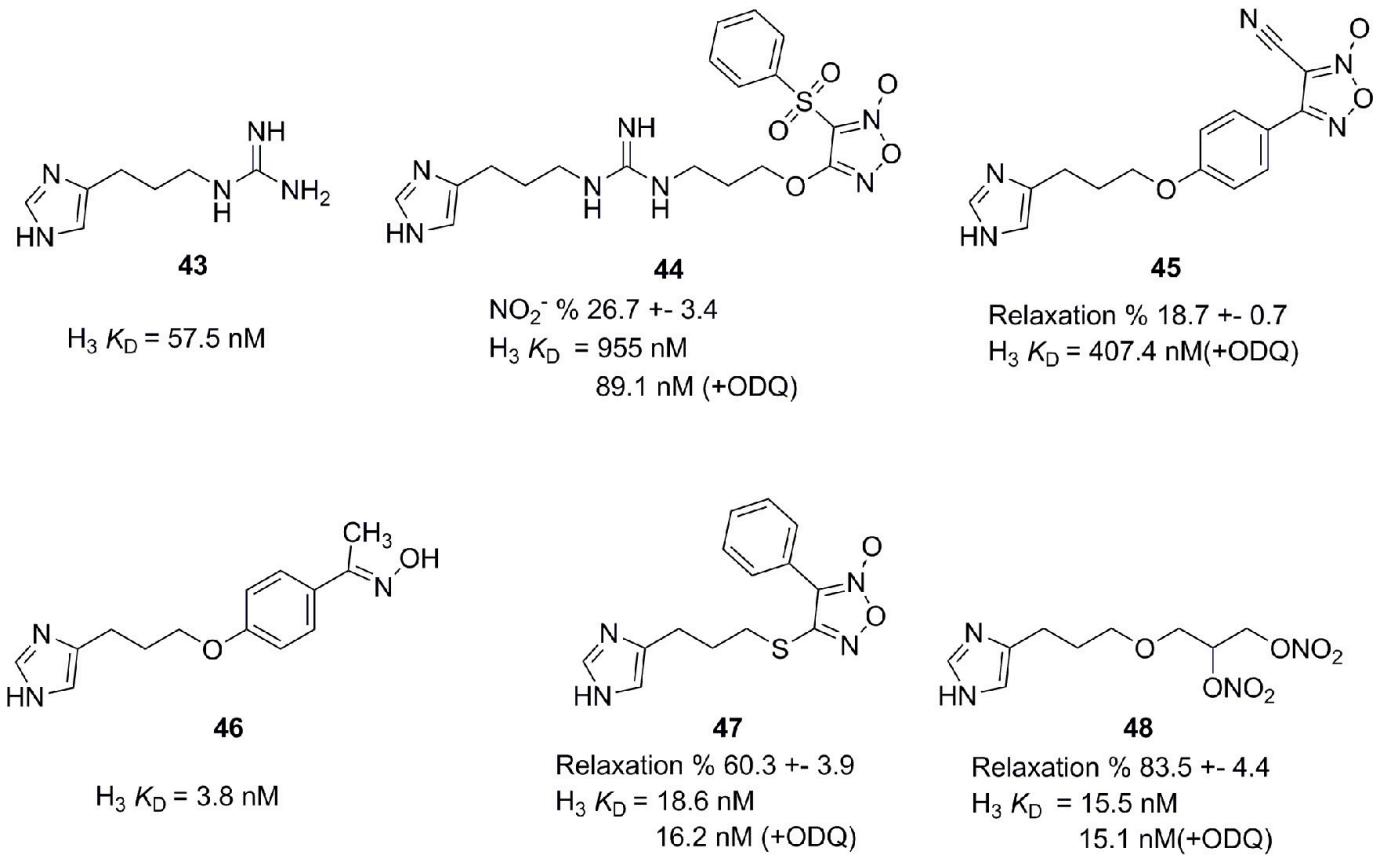
**Structures and biological activities of selected H_3_R/NO-donor ligands**.

Nitric oxide (NO) is an endogenous messenger, displaying a variety of actions in our body (Kerwin et al., 1995). NO is a key messenger in cardiovascular, immune, central, and peripheral nervous systems (Szabo, 2010). Released in the CNS after stimulation of excitatory NMDA, it diffuses in the adjacent presynaptic nerve terminal and astrocytes. There it activates the soluble guanylate cyclase (sGC) implying a number of physiological roles like gastro-protective effect, control of food intake and learning and formation of memory. H_3_R antagonists have also shown positive effects concerning learning and memory (Miyazaki et al., 1997; Komater et al., 2005). Combining H_3_R antagonists with NO-releasing moiety could synergistically contribute to a curative effect in pathologies like memory and learning disorders. Bertinaria et al. synthesized and tested some H_3_R antagonists with NO-donor properties by coupling H_3_R antagonist SKF 91486 (**43**, Figure 12) with the furoxan system (1,2,5-oxadiazole 2-oxide), which is able to release NO under the action of thiol cofactors like cysteine (Schönafinger, 1999). Resulting compounds had similar or greater potency as SKF 91486 (**43**). Derivative **44** (Figure 12) showed additional NO-dependent muscle relaxation (Bertinaria, 2003; Bertinaria et al., 2003). Another potent compound **45** is derived from Imoproxifan **46** (Figure 12) by replacing the oxime moiety with a five-membered NO-donor furoxan ring (Tosco et al., 2005). As a further development, a new class of NO-donor H_3_R antagonists with non-basic (thio)ether linker and furoxan (**47**) or nitrooxy (**48**) NO-donor moieties is introduced (Figure 12). These compounds are more appropriate to enter the CNS due to a better lipophilic-hydrophilic balance (Tosco et al., 2004).

## H_3_R and different antiseizure pharmacophores

**Figure 13:**
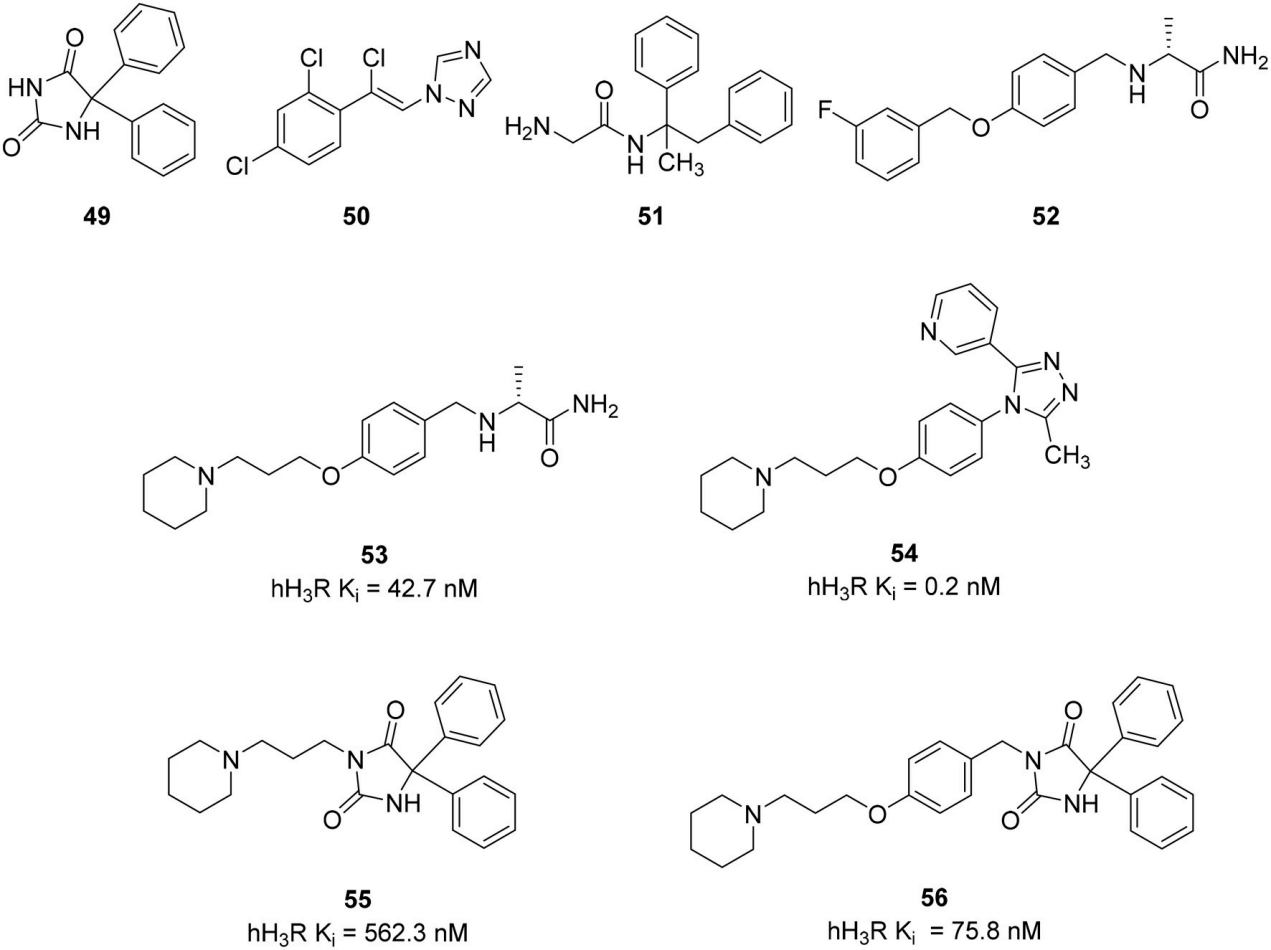
**Structures and biological activities of selected H_3_R/antiseizure ligands**.

Epilepsy is a common human brain disorder, affecting more than 60 million people worldwide. There is a need to discover an effective and safer antiepileptic drugs (AED) since Phenytoin (**49**) and recent AEDs like Loreclezole (**50**), Remacemide (**51**), and Safinamide (**52**) (Figure 13) only show efficacy within a maximum of 60–80% of patients and are responsible for many unwanted side-effects, such as headache, nausea, anorexia, ataxia, hepatotoxicity, drowsiness, gastrointestinal disturbance, gingival hyperplasia, attention deficit, und cognitive problems leading to additional discomfort (Sadek et al., 2014). There are indices for histamine receptors to improve the development of convulsions (Kasteleijn-Nolst Trenité et al., 2013). Seizure threshold can be increased and seizure susceptibility to electrically and chemically induced seizures can be decreased *via* activation of the central histaminergic system (Zhu et al., 2007; Bhowmik et al., 2012). Pitolisant has been tested in clinical trial phase II for patients suffering from photosensitive epilepsy. Supported by these results Sadek et al. designed some multiple-target ligands by combining the known 3-piperidinopropoxy or (3-piperidinopropoxy)aryl H_3_R pharmacophore with different AEDs on the market (**49–52**) leading to a small series of compounds (**53–56**, Figure 13; Sadek et al., 2014). These compounds showed moderate to good affinity to H_3_R with K_i_ values in the range of 562–0.24 nM and were tested *in vivo* for their anticonvulsive effect against maximum electroshock (MES)-induced and pentylenetetrazole (PTZ)-kindled convulsions in rats having phenytoin (**55**) as the reference AED. Surprisingly the compound with the lowest *in vitro* potency (**55**) was the only one to show the ability to reduce convulsions in both *in vivo* models being administered at 10 mg/kg intraperitoneally. Still the results are controversial and need new epilepsy models to elucidate the pharmacological profile of the current multiple targeting class in order to develop suitable and clinically useful AEDs (Bertinaria, 2003).

## Conclusion

Several combinations of different H_3_R pharmacophores with pharmacophoric elements of other histamine subtypes, other aminergic GPCRs, other transporters, other enzymes, and other disease-modifying elements have been described. The increasing knowledge on the complex interaction of the different signaling pathways as well as on the complex mechanism of central disorders, give promises for the development of optimized drugs with synergistic pharmacological properties at multiple targets and also reduced side effects. The different leads for MTDLs described here, are very early or at best preclinical candidates. Therefore, a lot of work on improvements has to be performed before these designed multiple targeting approaches will get into clinical trials.

## Author contributions

All authors listed, have made substantial, direct and intellectual contribution to the work, and approved it for publication.

### Conflict of interest statement

The authors declare that the research was conducted in the absence of any commercial or financial relationships that could be construed as a potential conflict of interest.
